# Novel cetacean morbillivirus in a rare Fraser’s dolphin (*Lagenodelphis hosei*) stranding from Maui, Hawai‘i

**DOI:** 10.1038/s41598-021-94460-6

**Published:** 2021-08-09

**Authors:** Kristi L. West, Ilse Silva-Krott, Nelmarie Landrau-Giovannetti, Dave Rotstein, Jeremiah Saliki, Stephen Raverty, Ole Nielsen, Vsevolod L. Popov, Nicole Davis, William A. Walker, Kuttichantran Subramaniam, Thomas B. Waltzek

**Affiliations:** 1 Hawai‘i Institute of Marine Biology, University of Hawai‘i at Manoa, Kaneohe, HI USA; 2 Human Nutrition Food and Animal Sciences, College of Tropical Agriculture and Human Resources, University of Hawai‘i at Manoa, Honolulu, HI USA; 3 Department of Infectious Diseases and Immunology, College of Veterinary Medicine, University of Florida, Gainesville, FL USA; 4 Marine Mammal Pathology Services, Olney, MD USA; 5 Oklahoma Animal Disease Diagnostic Laboratory, College of Veterinary Medicine, Oklahoma State University, Stillwater, OK USA; 6 Animal Health Center, British Columbia Ministry of Agriculture, Abbotsford, BC Canada; 7 Department of Fisheries and Oceans Canada, Winnipeg, MB Canada; 8 Department of Pathology, The University of Texas Medical Branch, Galveston, TX USA; 9 Pacific Islands Regional Office, National Marine Fisheries Service, Honolulu, HI USA; 10 Marine Mammal Laboratory, National Marine Fisheries Service, Seattle, WA USA

**Keywords:** Pathogens, Infectious diseases, Zoology, Diseases

## Abstract

Cetacean morbillivirus (CeMV) is a global threat to cetaceans. We report a novel morbillivirus from a Fraser’s dolphin (*Lagenodelphis hosei*) that stranded in Maui, Hawaii in 2018 that is dissimilar to the beaked whale morbillivirus previously identified from Hawaii and to other CeMV strains. Histopathological findings included intranuclear inclusions in bile duct epithelium, lymphoid depletion, rare syncytial cells and non-suppurative meningitis. Cerebellum and lung tissue homogenates were inoculated onto Vero.DogSLAMtag cells for virus isolation and cytopathic effects were observed, resulting in the formation of multinucleated giant cells (i.e., syncytia). Transmission electron microscopy of infected cell cultures also revealed syncytial cells with intracytoplasmic and intranuclear inclusions of viral nucleocapsids, consistent with the ultrastructure of a morbillivirus. Samples of the cerebellum, lung, liver, spleen and lymph nodes were positive for morbillivirus using a reverse transcription-polymerase chain reaction. The resulting 559 bp *L* gene sequence had the highest nucleotide identity (77.3%) to porpoise morbillivirus from Northern Ireland and the Netherlands. The resulting 248 bp *P* gene had the highest nucleotide identity to porpoise morbillivirus in Northern Ireland and the Netherlands and to a stranded Guiana dolphin (*Sotalia guianensis*) in Brazil (66.9%). As Fraser’s dolphins are a pelagic species that infrequently strand, a novel strain of CeMV may be circulating in the central Pacific that could have additional population impacts through transmission to other small island-associated cetacean species.

## Introduction

*Cetacean morbillivirus* (CeMV) of the family *Paramyxoviridae* and the genus *Morbillivirus*, has been associated with lethal outbreaks worldwide and represents one of the greatest infectious disease threats to cetaceans. Three strains of CeMV are well recognized, including the first description of porpoise morbillivirus (PMV) in two harbor porpoises (*Phocoena phocoena)*^1^*,* dolphin morbillivirus (DMV) that was identified for the first time from striped dolphins (*Stenella coeruleoalba)*^2^ and pilot whale morbillivirus (PWMV) that was initially identified from a long-finned pilot whale (*Globicephala melas)*^3^. More recently, three other strains of CeMV have been described, two from the Southern Hemisphere (Western Australia and Brazil) and the beaked whale morbillivirus (BWMV) from Hawaii^4–6^. These six strains have been clustered into two lineages; the CeMV-1 lineage that includes DMV, PMV, PWMV and BWMV and the CeMV-2 lineage that is represented by the two Southern hemisphere strains^7^. One of the CeMV-2 strains was first described in two Indo-Pacific bottlenose dolphins (*Tursiops aduncus)* from Western Australia^5^ and was later believed to be a major contributor to an unusual mortality event in Southern Australia Indo-Pacific bottlenose dolphins in 2013 where CeMV nucleotide similarity was 99.7% when compared to the CeMV strain initially described from Western Australia^8^. The other novel Southern hemisphere CeMV-2 strain was first described from a Guiana dolphin (*Sotalia guianensis)* in Brazil^4^. The Guiana dolphin cetacean morbillivirus (GDMV) was later linked to an unusual mortality event in Brazil where over 200 Guiana dolphins died^9^ and most recently a CeMV identified in 3 stranded Southern right whales (*Eubalaena australis*) in Brazil appeared to be similar to GDMV^10^.

In Hawaiian waters, the BWMV strain identified in a Longman’s beaked whale (*Indopacetus pacificus*) that stranded in 2010 off of Maui, Hawaii, represented the first report of CeMV in the central Pacific^6^. A retrospective screening of archived tissues from Pacific Island stranded cetaceans found the presence of BWMV in 12 different cetacean species, but no Fraser’s dolphins were represented among the 18 cetacean species tested. The retrospective study indicated 24% prevalence of BWMV in at least one tissue by polymerase chain reaction (PCR) in fresh carcasses although in many cases accompanying pathology was not observed^11^. Full genome sequencing of the BWMV identified from Hawaii confirms that BWMV represents a separate strain of CeMV among the CeMV-1 lineage^12^.

Understanding the threat posed by CeMV to Hawaiian cetaceans is critical as many species represent small, island associated populations where a morbillivirus outbreak could be devastating. The present study describes a novel morbillivirus from a stranded Fraser’s dolphin in Hawaii that was dissimilar when the partial sequence was compared to the RNA-dependent RNA polymerase (*L *gene) and phosphoprotein (*P* gene) sequences of other morbillivirus strains. Strandings of Fraser’s dolphins in Hawaii are rare with few reports of this species close to shore^13–15^.

## Results

Prey identification was conducted for the stranded Fraser’s dolphin. Stomach contents included 13 lower beaks from cephalopods and a small piece of green fishing line (approximately 31 mm in length). The 13 lower beaks represented five different species from four cephalopod families. The family *Histioteuthidae* represented the greatest contribution to diet of this individual, with 53.8% contribution to diet by abundance and 74% by mass. The family *Mastigoteuthidae* contributed 23.1% by abundance to the diet and 13% by mass. Other cephalopod families identified among the stomach contents included *Enoploteuthidae* and *Neoteuthidae* which contributed less than 10% by prey abundance and by prey mass.

**Figure 1:**
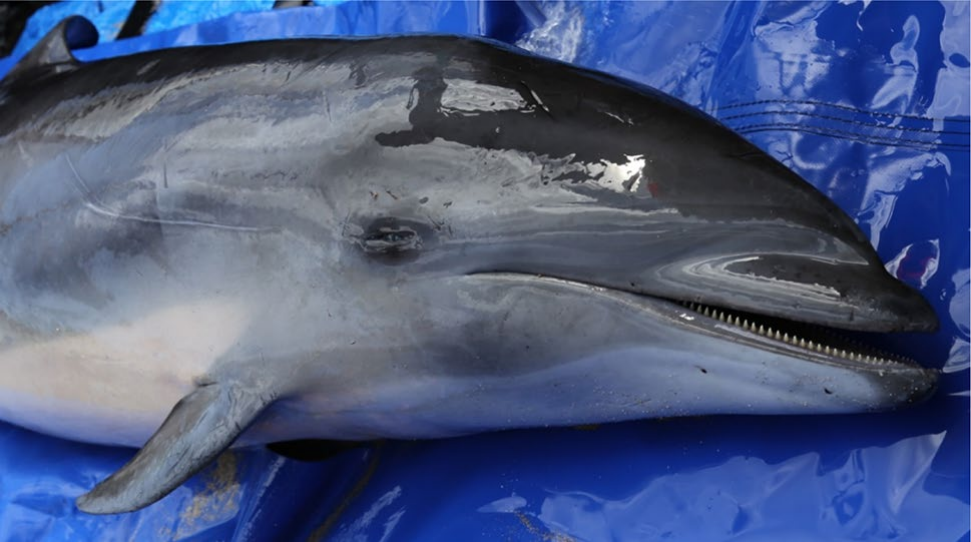
The sub-adult male Fraser’s dolphin (*Lagenodelphis hosei*) that stranded at Olowalu, Maui, Hawaii. Photo courtesy of Cindy Kern.

**Figure 2:**
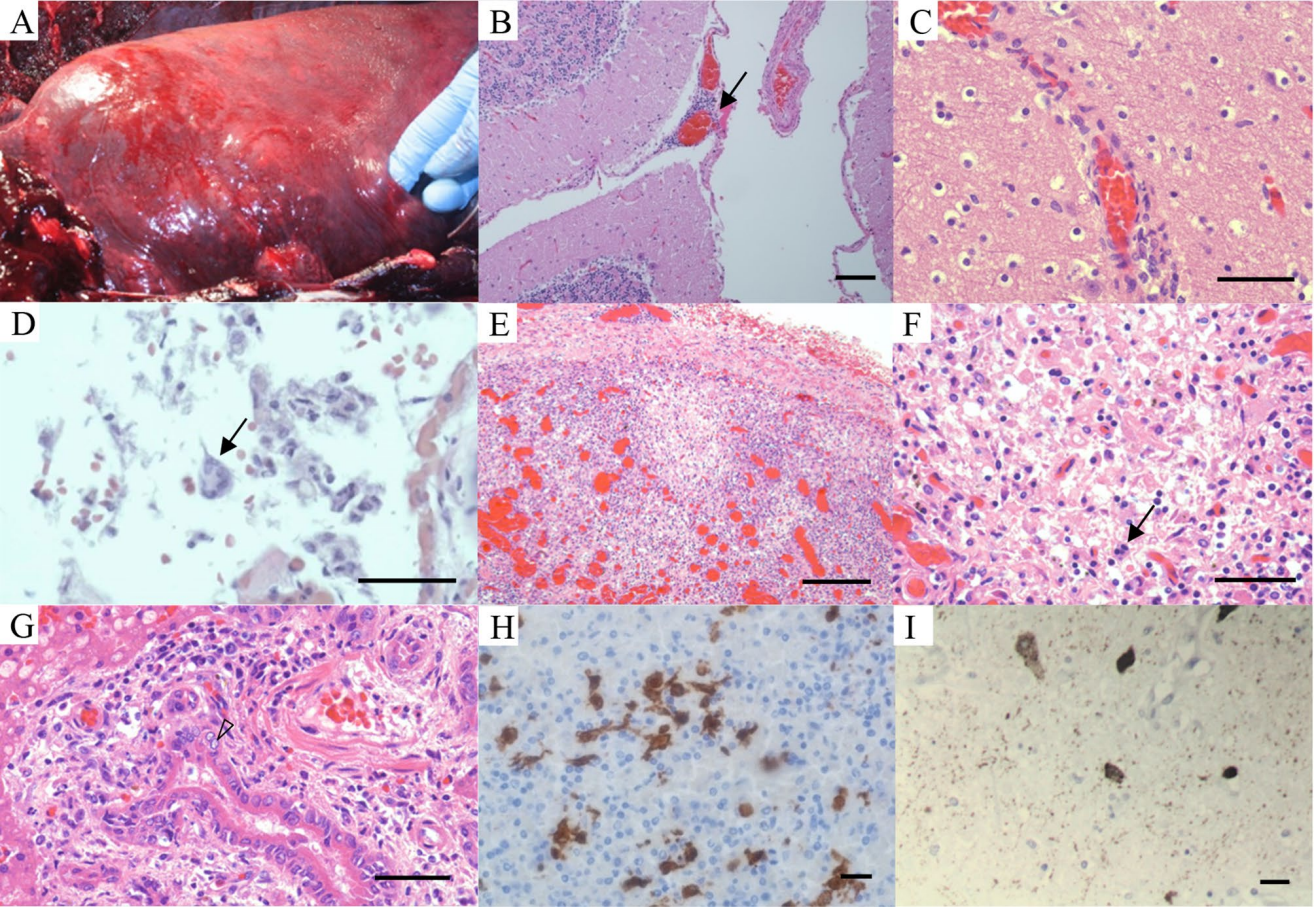
(**A**) Left lung. There are firm nodules present throughout the pulmonary parenchyma. The nodules may also be related to parasitism; (**B**) Meninges. H&E stain: Lymphocytic infiltrates within the meninges (arrow). Bar = 100 µm; (**C**) Cerebrum. H&E stain: Perivascular lymphocytic cuffing. Bar = 2 mm; (**D**) Lung. H&E stain: Alveolar spaces contain sloughed Type II pneumocytes, erythrocytes and rare syncytial cells (arrow). Vessels are congested. Bar = 500 µm; (**E**) Lymph node. H& E stain: Lymphoid follicle depletion. Bar = 2 mm; (**F**) Lymph node. H&E stain: Germinal center necrosis. Syncytium (arrow). Bar = 500 µm; (**G**) Bile duct. H&E stain: Portal hepatitis with biliary hyperplasia and viral nuclear inclusions in biliary epithelial cells (arrow head) Bar = 500 µm; (**H**) Lymph node. Morbillivirus immunohistochemical stain. Bar = 20 µm; (**I**) Brain. Morbillivirus immunohistochemical stain: Immunoreactivity within neuronal perikarya. Bar = 500 µm;

The stranded dolphin was in good body condition, had a total body length of 2.04 m, weighed 183.5 kg and was an immature male (Fig. 1). Gross necropsy findings included cookie cutter shark bites and abrasions up to 30 cm in length along the lateral sides of the body that are likely associated with coming across reef or rocks during the stranding event. Multiple abrasions were also documented on the dorsal fluke surface. The left eye was intact, but a bleeding open wound was observed near the medial canthus that may have been due to bird scavenging of the carcass prior to recovery. A minimum of six small crustacean parasites (whale lice) were observed near the blowhole opening. The deeper blubber was bruised extending from the left cranial to the axillary region with moderate hemorrhage observed at the blubber-muscular interface. The left lung weighed 1188 g and the right lung weighed 1292 g. The lungs had a mottled surface, with areas of consolidation (1–2 cm in diameter) (Fig. 2A). Areas of consolidation revealed dark red to tan firm tissue around small bronchi in cross-section. The left lung marginal lymph node was enlarged and had an irregular surface. The trachea and main bronchi contained small amounts of red-tinged foam. Serous membranes in the thorax and abdomen were unremarkable. The liver weighed 2155 g and an enhanced lobular pattern was observed on cut surface. The spinal fluid was blood-tinged, and meningeal blood vessels were dilated.

### Histopathology findings and initial diagnostics

Histopathological findings of note were observed in the lymph nodes, spleen, brain, lung and liver. Lymphocytes and plasma cells (non-suppurative inflammation) were sprinkled within the meninges and cerebral blood vessels exhibited mild lymphocytic cuffing (Fig. 2B,C). Rare syncytial cells were observed in alveoli intermixed with erythrocytes, macrophages and scant proteinaceous fluid. Pulmonary blood vessels were dilated (Fig. 2D). The liver had scattered periportal hepatocellular necrosis and portal tract expansion with moderate hyperplasia of bile ducts and small mixed inflammatory infiltrates were observed. Lymphoid organs were depleted and there was germinal center necrosis with macrophages and syncytial cells present (Fig. 2E,F). Bile duct epithelial cells had occasional large clear nuclei with faint eosinophilic intranuclear inclusions (Fig. 2G).

### Immunohistochemistry

Morbilliviral antigen was detected in the cerebrum, cerebellum, spleen, lung, kidney and lymph nodes (Table 1). Immunoreactivity was observed in lymphocytes and macrophages within lymph nodes (Fig. 2H) and the spleen; in neurons and glial cells in the brain (Fig. 2I); pneumocytes, bronchial mucosal epithelial cells, macrophages and syncytial cells in the lung; and tubular epithelial cells in the kidney. Staining was diffuse and intense. Staining was intracytoplasmic and intranuclear.

**Table 1:** Fraser dolphin tissue types tested for the presence of morbillivirus where (-) indicates that the tissue type was not examined.

Tissue Type:	*Morbillivirus* (IHC)	*Morbillivirus* (RT-PCR)	*Morbillivirus* (Isolation)
Cerebrum	*Positive*	*-*	Negative
Cerebellum	*Positive*	*Positive*	*Positive*
Spleen	*Positive*	*Positive*	-
Liver	-	*Positive*	-
R. Lung	-	-	*Positive*
L. Lung	*Positive*	*Positive*	*Positive*
Kidney	*Positive*	-	-
L. hilar lymph node	-	*Positive*	Negative
Mediastinal lymph node	-	*Positive*	-
R. pre-scapular lymph node	-	*Positive*	-
Mesenteric lymph node	*Positive*	-	Negative
Retroperitoneal lymph node	-	-	Negative
Unidentified lymph node 1	*Positive*	-	-
Unidentified lymph node 2	*Positive*	-	-
Unidentified lymph node 3	*Positive*	-	-

### Morbillivirus isolation

**Figure 3:**
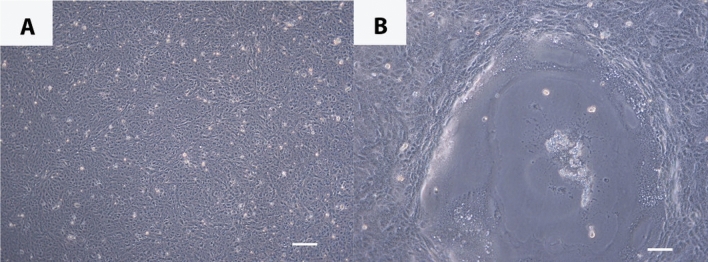
(**A**) Vero.DogSLAMtag control cells. Bar = 200 µm; (**B**) Vero.DogSLAMtag infected with Fraser’s dolphin cerebellum 8 days post infection showing large syncytium. Bar = 200 µm.

Vero.DogSLAMtag cells were chosen to attempt virus isolation of CeMV from cerebrum, cerebellum, right and left lungs, left hilar lymph node, mediastinal lymph node, retroperitoneal lymph node and mesenteric lymph node tissues that were collected from the stranded Fraser’s dolphin. Seven days post infection no cytopathic effect (CPE) was observed and the flasks were passaged at a ratio of 1–5 but 24 h later small plaques developed in the flasks inoculated with both A and B samples of cerebellum (Fig. 3a,b). Plaques contained many multinucleated giant cells or syncytia, a finding consistent with morbillivirus infection. Over the next few days, similar foci developed in both right and left lung flasks. This effect could be duplicated with dilution (1:500), filtration through a 0.45 micron filter and passage onto fresh cells. No cytopathic effect was seen in flasks from the other samples including the cell controls.

### Transmission electron microscopy

**Figure 4:**
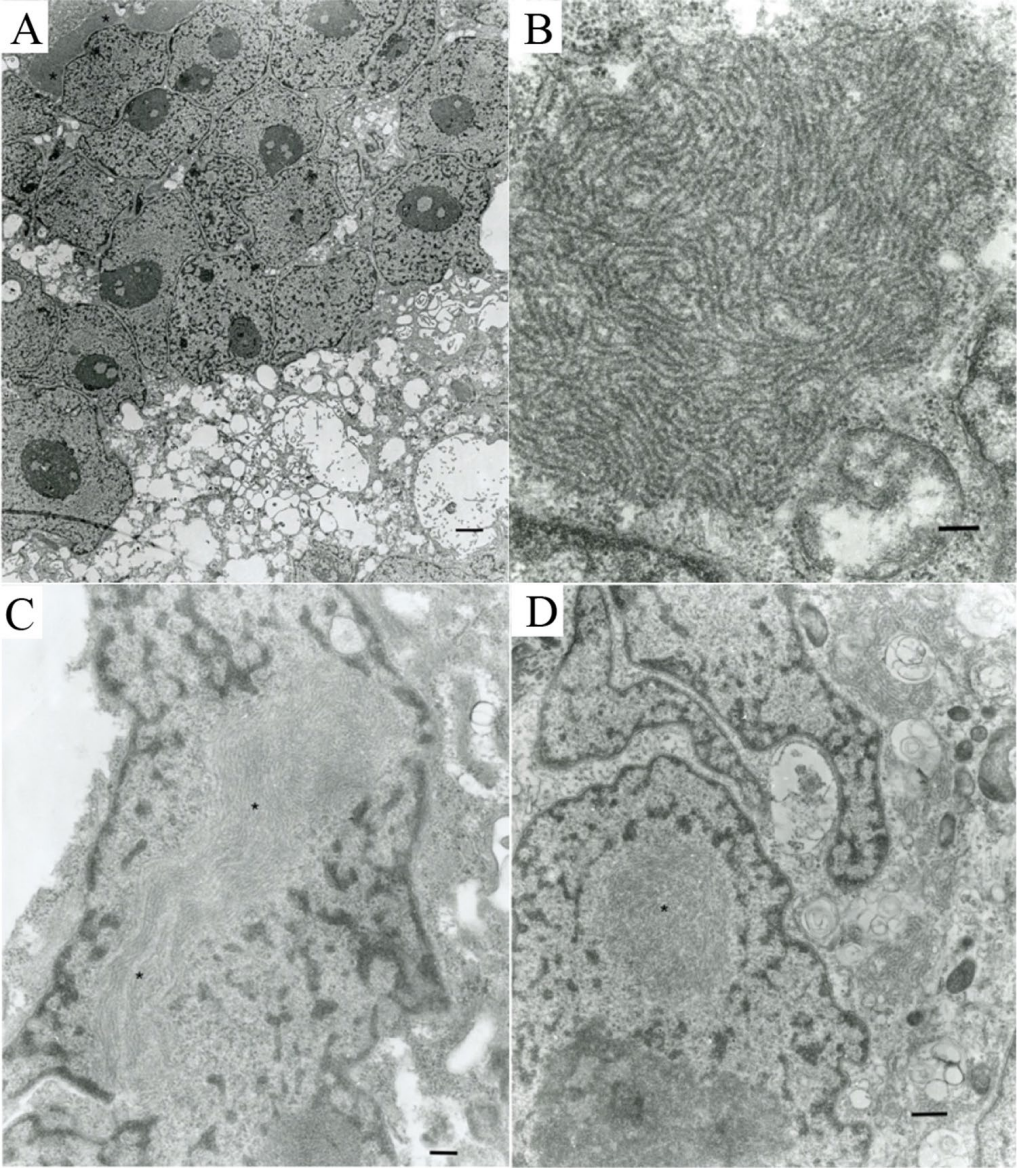
(**A**) A portion of a syncytium with multiple nuclei and a morbillivirus nucleocapsid cytosolic inclusion (upper left corner, asterisk). The surface of the monolayer (bottom) looks “foamy” due to multiple projections of plasmalemma forming multiple “vacuoles’. Bar = 2 µm; (**B**) A fragment of intracytosolic nucleocapsid inclusion with multiple (spiral) strands ~ 20 nm in diameter. A portion of the nucleus is in the left lower corner. Bar = 200 nm; (**C**) Large intranuclear inclusion of morbillivirus nucleocapsid (asterisk) in an attached cell in the monolayer 7 days post-infection (dpi). Bar = 200 nm; (**D**) Intranuclear inclusion of nucleocapsid in a syncytium (asterisk). Portions of two other nuclei at the top of an infected one. Attached cells 7 dpi. Bar = 500 nm.

In ultrathin sections of infected Vero.DogSLAMtag monolayers giant cells with multiple nuclei (syncytia) were often observed (Fig. 4A). Most times their surface was “foamy” with multiple projections of cell membrane forming “vacuoles” (Fig. 4A). Those syncytia often contained cytoplasmic inclusions of viral nucleocapsids (Fig. 4A) consisting of intertwined strands ~ 20 nm in diameter (Fig. 4B), also suggestive of morbillivirus infection. At later time points of infection, those inclusions could be observed at the cell membrane but budding of the virus into the extracellular space in attached or free-floating cells was not observed. At later stages of infection intranuclear inclusions of viral nucleocapsids were regularly observed (Fig. 4C,D).

### Morbillivirus detection by RT-PCR

**Figure 5:**
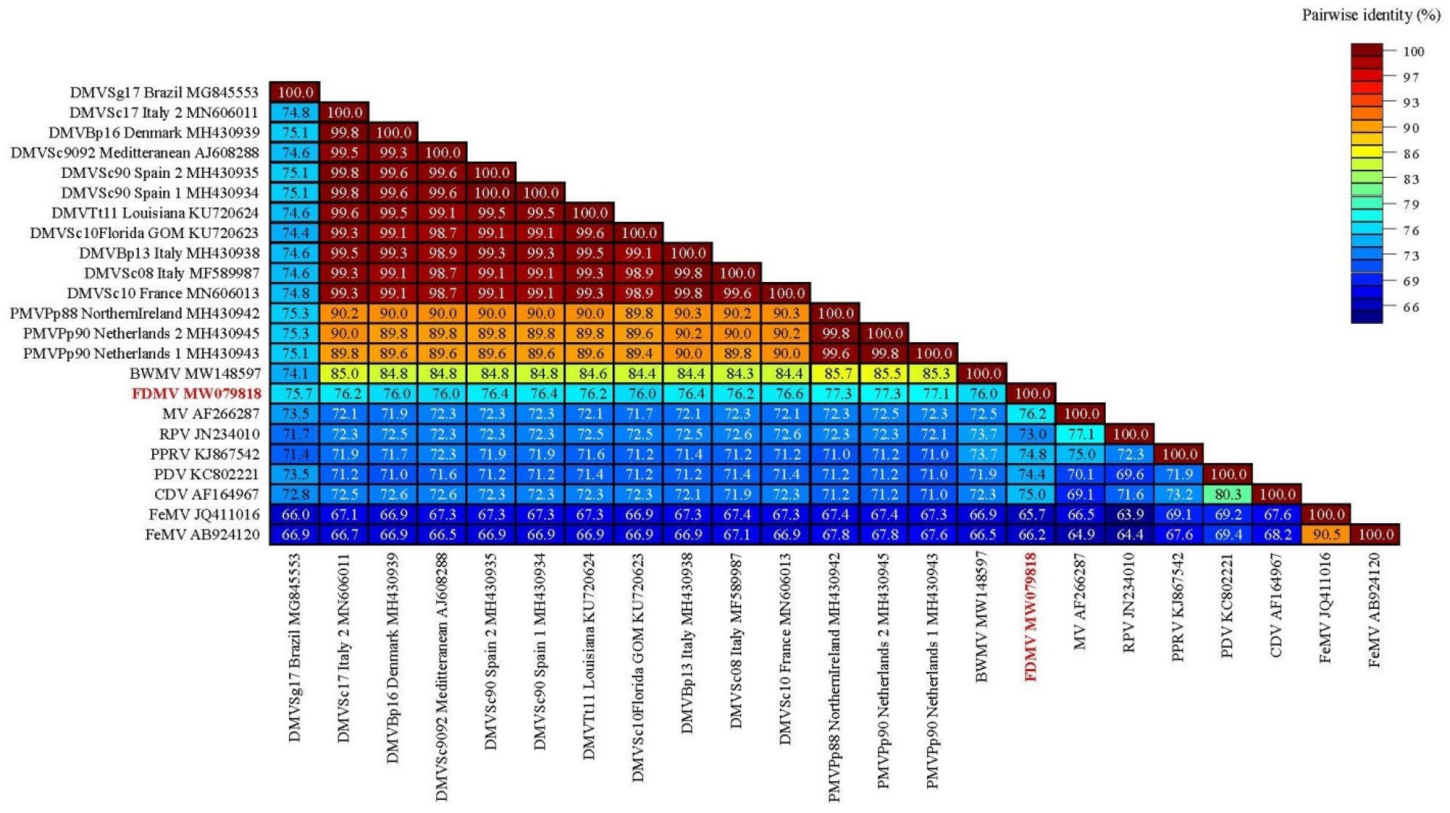
Genetic analysis of 23 partial nucleotide sequences of the *L* gene. The final data set contained 559 characters including gaps. Values are expressed as a percentage of identity. *BWMV* beaked whale morbillivirus; *FDMV* Fraser’s dolphin morbillivirus, *PWMV* pilot whale morbillivirus; *PMV* porpoise morbillivirus; *GDMV* Guiana dolphin morbillivirus; *DMV* dolphin morbillivirus; *MeV* measles virus; *RPV* rinderpest virus; *PPRV* peste-des-petits-ruminants virus; *PDV* phocine distemper virus; *CDV* canine distemper virus; *FeMV* feline morbillivirus.

Positive tissues by RT-PCR included the cerebellum, spleen, liver, left lung, mediastinal lymph node, right pre-scapular lymph node and the left hilar lymph node (Table 1). The partial *L* gene sequence (559 bp) and partial *P* gene sequence (248 bp) obtained from the Fraser’s dolphin were compared with *L* gene and *P* gene sequences of other morbilliviruses using the Sequence Demarcation Tool v1.2^16^, with the MAFFT alignment option implemented (Fig. 5). The partial *L* gene sequence of the Fraser’s dolphin morbillivirus displayed highest nucleotide identity (77.3%) to a PMV from harbor porpoises that stranded on the coast of Northern Ireland in 1988^17^ and in the Dutch Waddensea (North Sea-Netherlands) in 1990^18^. The partial *P* gene displayed highest nucleotide identity (66.9%) to a PMV from one harbor porpoise that stranded on the coast of Northern Ireland in 1988 ^17^, two that stranded in the Dutch Waddensea (North Sea-Netherlands) in 1990^18^ and from a 2017 mass die-off of Guiana dolphins in Brazil^9^.

## Discussion

This rare Fraser’s dolphin stranding provides insight into the biology and ecology of a poorly known species in the central Pacific. This is the first report of Fraser’s dolphin diet composition in Hawaiian waters where only cephalopod prey remains were present among the stomach contents. In other regions, including the Eastern Tropical Pacific, Taiwan and the Philippines, Fraser’s dolphin diet of mesopelagic fishes, cephalopods and crustaceans has been described^19–22^. It could be argued that the diet described from a single stranded individual may not reflect normal feeding behavior. However, there is considerable overlap in the mesopelagic taxa of cephalopods observed in the stomach of the Hawaii individual and the Fraser’s dolphins from the Philippines and Taiwan ^[19.22]^ leading us to believe that this sample is, at least in part, representative of the diet of Fraser’s dolphins inhabiting the central Pacific.

The major significance of this Fraser’s dolphin stranding is the discovery of a novel CeMV in Hawaiian waters that we have described as Fraser’s dolphin morbillivirus. The lesions documented in multiple body systems were subacute and death may have possibly resulted from neurologic and hepatic dysfunction. Although non-specific, gross and histopathological findings are consistent with morbillivirus infection in cetaceans^23^ including lymphoid depletion and syncytial cells in the lung and lymph node. Viral inclusions were observed solely in the bile duct epithelium. Eosinophilic intranuclear inclusions in bile duct epithelia are described in morbillivirus infection of striped dolphins and were seen in this case^24^. Morbillivirus-associated non-suppurative meningoencephalitis was observed without neuronal changes; however, neurons exhibited immunoreactivity with immunohistochemical stains (Fig. 2I). Co-infection or secondary infection with other viral, bacterial, protozoal, and fungal pathogens are common with CeMV infections and can also contribute to death^25^. A stranded neonate sperm whale that stranded on Oahu in 2011 was co-infected with *Brucella* and morbillivirus^26^. A Longman’s beaked whale stranded in Maui in 2010 diagnosed with CeMV was tri-infected with an alphaherpesvirus and a novel circovirus^6,27^. *Brucella*, *Toxoplasma gondii* and herpesvirus were not detected during an intial diagnostic screen of this individual.

**Figure 6:**
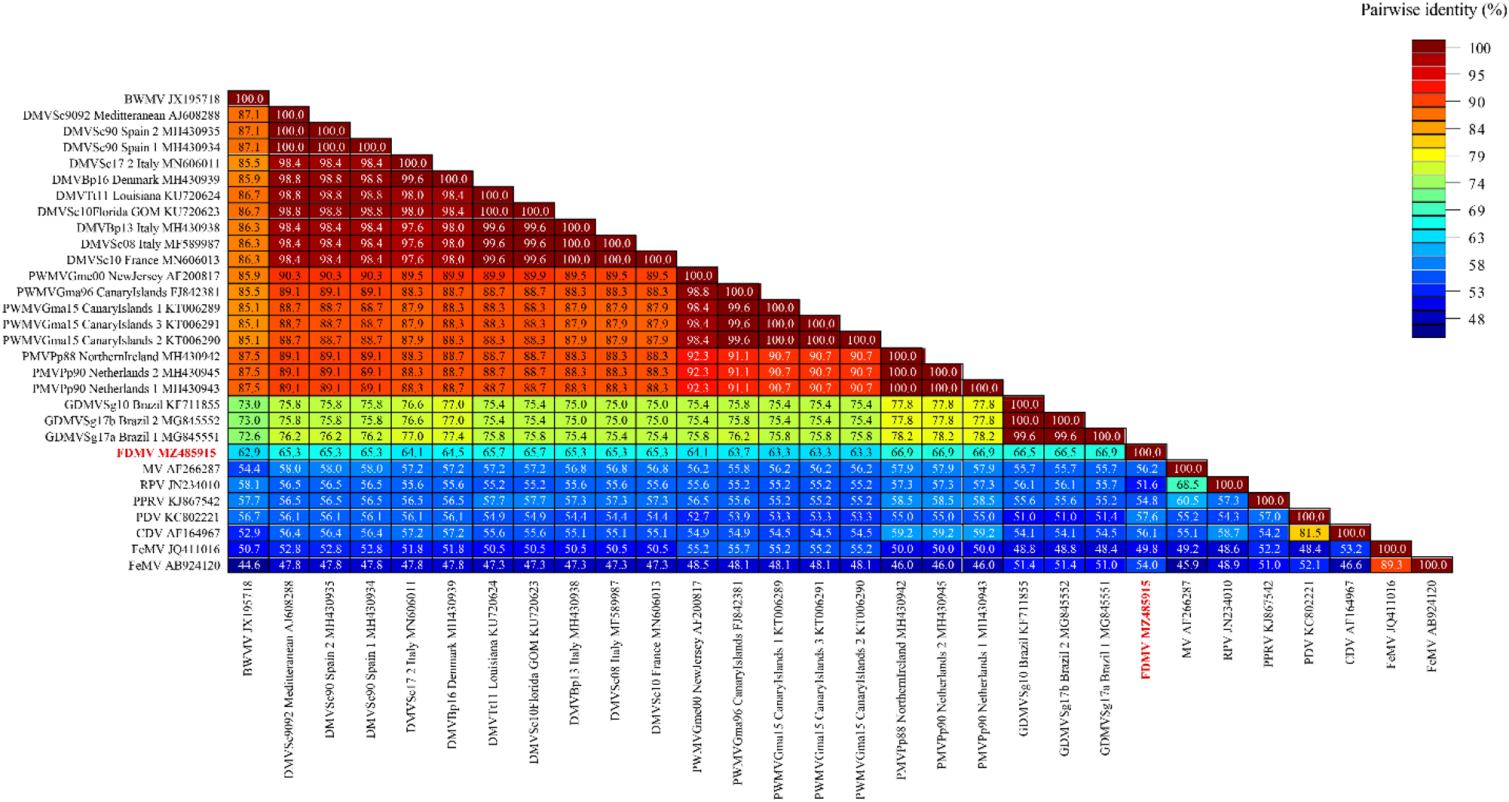
Genetic analysis of 30 partial nucleotide sequences of the *P* gene. The final data set contained 248 characters including gaps. Values are expressed as a percentage of identity. *BWMV* beaked whale morbillivirus; *FDMV* Fraser’s dolphin morbillivirus; *PWMV* pilot whale morbillivirus; *PMV* porpoise morbillivirus; *GDMV* Guiana dolphin morbillivirus; *DMV* dolphin morbillivirus; *MeV* measles virus; *RPV* rinderpest virus; *PPRV* peste-des-petits-ruminants virus; *PDV* phocine distemper virus; *CDV* canine distemper virus; *FeMV* feline morbillivirus.

**Figure 7:**
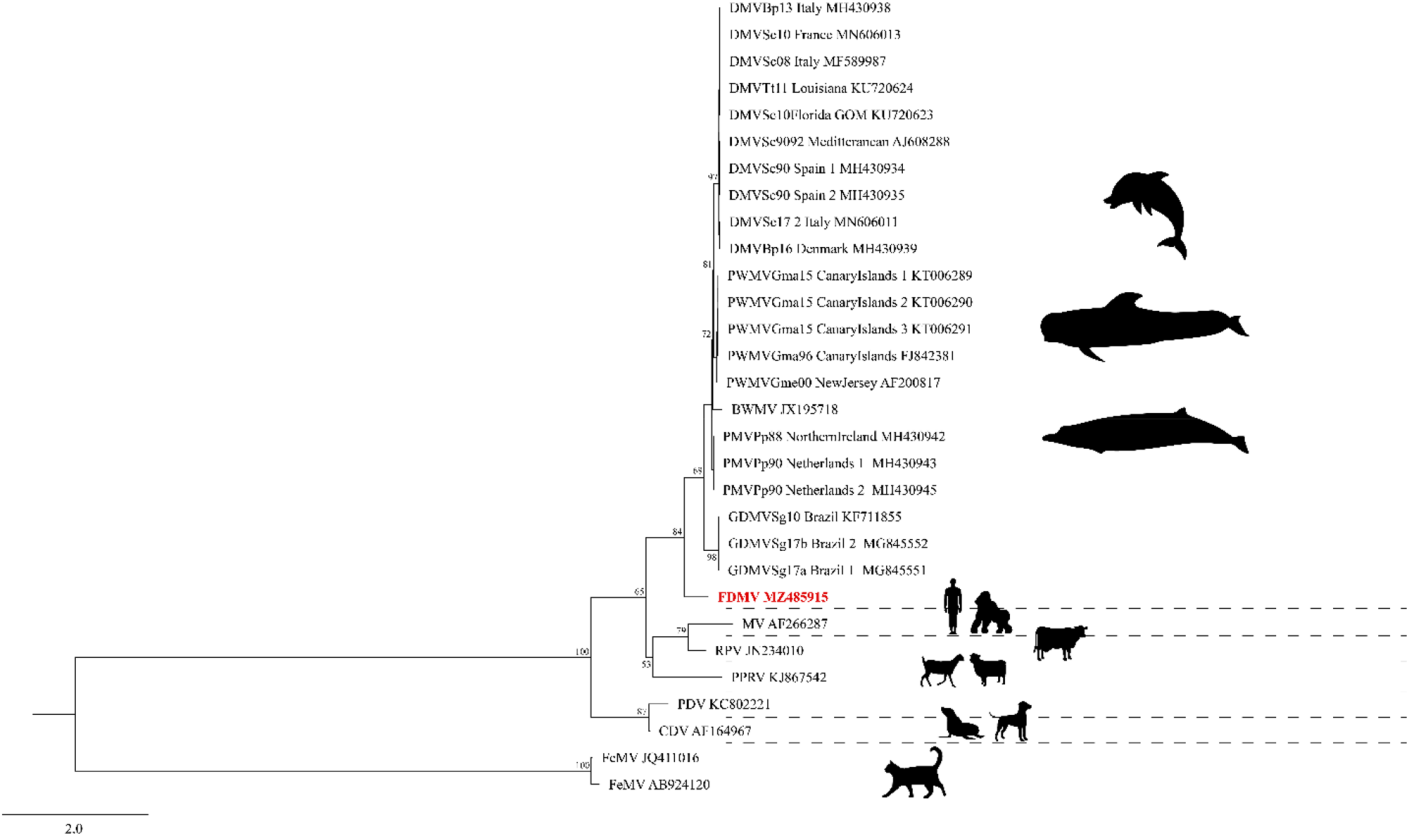
Maximum Likelihood (ML) phylogenetic analysis based on the partial nucleotide (nt) sequences of the *P* gene. Each sequence is denoted by its strain/isolate name, geographic area of stranding (where available), and GenBank accession number (where available). The phylogeny includes 30 nucleotide sequences with a total of 248 characters (including gaps) in the final dataset. IQ-TREE software (http://iqtree.cibiv.univie.ac.at/) was used to determine best-fit models and perform ML phylogenetic analysis with 1000 non-parametric bootstraps. The best-fit model for the alignment was TPM2 + G4TPM2u + G4, chosen based on Bayesian Information Criterion. *BWMV* beaked whale morbillivirus; *FDMV* Fraser’s dolphin morbillivirus; *PWMV* pilot whale morbillivirus; *PMV* porpoise morbillivirus; *GDMV* Guiana dolphin morbillivirus; *DMV* dolphin morbillivirus; *MeV* measles virus; *RPV* rinderpest virus; *PPRV* peste-des-petits-ruminants virus; *PDV* phocine distemper virus; *CDV* canine distemper virus; *FeMV* feline morbillivirus.

The partial sequences from both genes of the Fraser’s dolphin morbillivirus was surprisingly divergent from the sequences of the six known strains of CeMV^7^ and suggests that the Fraser’s dolphin morbillivirus is distinct (Fig. 5, 6, 7 and Table 2). Despite being described from Hawaiian waters, the nucleotide dissimilarity between the Fraser’s dolphin morbillivirus and the BWMV previously described in 12 species of cetaceans from Hawaii was considerable^6,11^. The partial Fraser’s dolphin morbillivirus *L* gene sequence only indicated 76% similarity when compared to BWMV^12^. The partial *P* gene of the Fraser’s dolphin morbillivirus was also relatively dissimilar to the Southern Hemisphere CeMV strain from Brazil indicating nucleotide similarity of only 66.5% and 66.9% to Brazil^12^. The only *L* gene sequence data for the Southern Hemisphere was a Brazilian strain (GenBank Accession No. MG845553) that indicated a 75.7% nucleotide similarity. Even the highest nucleotide identity of 77.3% between morbillivirus sequences from the Fraser’s dolphin and the harbor porpoise in Northern Ireland and the Netherlands is lower than initial comparisons between the BWMV described in Hawaiian waters and the recognized DMV, PMV and PMMV strains at the time of discovery of morbillivirus in the central Pacific (83.9–88.7% nucleotide similarity depending on *P* or *N* gene comparisons)^6,11^. Although we only determined the partial *L* gene (~ 559 bp) and *P* gene sequences (248 bp), our results suggest the presence of a novel morbillivirus in Hawaiian waters could be circulating in Fraser’s dolphins in the central Pacific. These findings emphasize the importance of full genome sequencing of the novel Fraser’s dolphin morbillivirus which is currently underway to address how this compares with other known strains of CeMV.

**Table 2:** Virus names, GenBank accession numbers, target gene and references for the morbilliviruses used in the genetic analysis of the *L* and *P* gene.

Virus name (virus abbreviation)	Common name	Scientific name	Gene	Accession number
USA Dolphin morbillivirus (DMVTt11_Louisiana)^28^	Bottlenose dolphin	*Tursiops truncatus*	Both	KU720624
USA Dolphin morbillivirus (DMVSc10Florida_GOM)^28^	Striped dolphin	*Stenella coeruleoalba*	Both	KU720623
Denmark Dolphin morbillivirus (DMVBp16_Denmark)^17^	Fin whale	*Balaenoptera physalus*	Both	MH430939
Netherlands Porpoise morbillivirus (PMVPp90_Netherlands(1))^17^	Harbor porpoise	*Phocoena phocoena*	Both	MH430943
Netherlands Porpoise morbillivirus (PMVPp90_Netherlands(2))^17^	Harbor porpoise	*Phocoena phocoena*	Both	MH430945
Spain Dolphin morbillivirus (DMVSc90_Spain(1))^17^	Striped dolphin	*Stenella coeruleoalba*	Both	MH430934
Spain Dolphin morbillivirus (DMVSc90_Spain(2))^17^	Striped dolphin	*Stenella coeruleoalba*	Both	MH430935
Italy Dolphin morbillivirus (DMVBp13_Italy)^17^	Fin whale	*Balaenoptera physalus*	Both	MH430938
Italy Dolphin morbillivirus (DMVSc08_Italy)^17^	Striped dolphin	*Stenella coeruleoalba*	Both	MF589987
Ireland Porpoise morbillivirus (PMVPp88_NorthernIreland)^17^	Harbor porpoise	*Phocoena phocoena*	Both	MH430942
Mediterranean Dolphin morbillivirus (DMVSc90-92_Meditteranean)^29^	Striped dolphin	*Stenella coeruleoalba*	Both	AJ608288
Brazil Dolphin morbillivirus (DMVSg17_Brazil)^9^	Guiana dolphin	*Sotalia guianensis*	*L* gene	MG845553
Italy Dolphin morbillivirus (DMVSc17_Italy(2))	Striped dolphin	*Stenella coeruleoalba*	Both	MN606011
France Dolphin morbillivirus (DMVSc10_France)	Striped dolphin	*Stenella coeruleoalba*	Both	MN606013
New Jersey Pilot Whale morbillivirus (PWMVGme00_NewJersey)^3^	Long-finned pilot whale	*Globicephalus melas*	*P* gene	AF200817
Canary Islands Pilot Whale morbillivirus (PWMVGma96_CanaryIslands)^30^	Short-finned pilot whale	*Globicephala macrorhynchus*	*P* gene	FJ842381
Canary Islands Pilot Whale morbillivirus (PWMVGma15_CanaryIslands(1))^31^	Short-finned pilot whale	*Globicephala macrorhynchus*	*P* gene	KT006289
Canary Islands Pilot Whale morbillivirus (PWMVGma15_CanaryIslands(3))^31^	Short-finned pilot whale	*Globicephala macrorhynchus*	*P* gene	KT006291
Canary Islands Pilot Whale morbillivirus (PWMVGma15_CanaryIslands(2))^31^	Short-finned pilot whale	*Globicephala macrorhynchus*	*P* gene	KT006290
Brazil Dolphin morbillivirus (GDMVSg10_Brazil)^4^	Guiana dolphin	*Sotalia guianensis*	*P* gene	KF711855
Brazil Dolphin morbillivirus (GDMVSg17_Brazil(2))^9^	Guiana dolphin	*Sotalia guianensis*	*P* gene	MG845552
Brazil Dolphin morbillivirus (GDMVSg17_Brazil(1))^9^	Guiana dolphin	*Sotalia guianensis*	*P* gene	MG845551
USA Dolphin morbillivirus (BWMVIp10_Hawaii)^12^	Longman’s beaked whale	*Indopacetus pacificus*	*L* gene	MW148597
USA Dolphin morbillivirus (BWMVIp10_Hawaii)^6^	Longman’s beaked whale	*Indopacetus pacificus*	*P* gene	JX195718
USA Dolphin morbillivirus (FDMVLh18_Hawaii)^12^	Fraser’s dolphin	*Lagenodelphis hosei*	*L* gene	MW079818
USA Dolphin morbillivirus (FDMVLh18_Hawaii)^12^	Fraser’s dolphin	*Lagenodelphis hosei*	*P* gene	MZ485915
Measles virus (MV)^32^	Edmonston vaccine strain	N/A	Both	AF266287
Rinderpest virus (RPV)^33^	Fusan vaccine strain	N/A	Both	JN234010
Peste des petits ruminants virus (PPRV)	Domestic goat	*Capra aegagrus hircus*	Both	KJ867542

Despite our repeated TEM studies, we never observed the Fraser’s dolphin morbillivirus budding into the extracellular spaces in vitro. This is in contrast to other morbilliviruses that bud into extracellular spaces, making it possible for virus transmission of complete virions by aerosol transmission^34^. Since complete virus particles were never seen, we hypothesize that virus transmission between Fraser’s dolphins may occur via the aerosolizing of infected syncytial cells coming in direct cell to cell contact with a susceptible new host. Cell to cell transmission by syncytia within the same host has been documented in measles infections^35^.

Fraser’s dolphins are a pelagic species that is poorly known from the world’s oceans. There are no previous reports of morbillivirus in Fraser’s dolphins from Hawaiian waters. In the greater United States seronegative results were obtained from 10 Fraser’s dolphins that mass stranded in Florida in 2003 and PMV seropositive results from 11 of 23 mass stranded Fraser’s dolphins in the Gulf of Mexico in 1994^36,37^. Additionally, three of four Fraser’s dolphins that had stranded in 1997 and 1999 along the coasts of Argentina and Brazil respectively, had antibodies against DMV^38^. This suggests that morbillivirus is likely endemic in Fraser’s dolphins from the Gulf of Mexico and the Southwest Atlantic and it is possible that a novel strain of morbillivirus is similarly circulating among this species in the central Pacific. Fraser’s dolphin strandings are extremely rare in Hawaiian waters, with the 2018 individual only the second confirmed stranding of this species in this region. A young Fraser’s dolphin was reported dead off Kauai in an advanced state of decomposition in 2004 and a possible newborn Fraser’s dolphin was reported dead stranded in 1976^15^. With such extreme rarity of stranding, we have not had the opportunity to date to test additional Fraser’s dolphins from the central Pacific for the presence of morbillivirus or morbillivirus antibodies.

At this time, the population impact of the novel Fraser’s dolphin morbillivirus on this species and the potential impact on other cetacean species inhabiting the central Pacific is unknown. Considering the highly social nature of cetacean groups and that different cetacean species interact, it is probable that the novel Fraser’s dolphin morbillivirus could be transmitted to other Hawaiian species. This is especially concerning as many of Hawaii’s cetacean stocks are small, island-associated, resident populations that may be particularly vulnerable to any reduction in population size because of an already low number of breeding individuals such as the endangered main Hawaiian Islands insular false killer whale *(Pseudorca crassidens)*, estimated at only 167 individuals^39^. Novel disease is considered a major hurdle to endangered population recovery and even poses the threat of extinction as demonstrated in a recent effort to model the impact of a morbillivirus outbreak on endangered Southern resident killer whales (*Orcinus orca*) from the Pacific Northwest^40^. Southern resident killer whales were determined to be highly vulnerable to a disease outbreak^40^; similarly, a novel disease outbreak among Hawaii’s endangered false killer whale population could have a disastrous impact. Melon-headed whales (*Peponocephala electra*) in Hawaii are also genetically and behaviorally distinct, with a Kohala resident population of approximately 500 individuals inhabiting shallower waters off the Big Island^41^. Fraser’s dolphins have only been sighted six times in Hawaiian waters in over two decades of survey effort, and on four of these occasions, this species was sighted near Hawaii Island with large groups of melon-headed whales and once with pilot whales that were part of the Kona resident stock (Baird, personal communication). The Kohala resident stock of melon-headed whales is frequently sighted interacting with other species of cetaceans, including rough-toothed dolphins (*Steno bredanensis)* and pilot whales (*Globicephala macrorhynchus)* that move among the Hawaiian islands and overlap with insular slope cetacean populations, which provides a potential route for widespread transmission among Hawaiian cetaceans. Spotted dolphins (*Stenella attenuata*), spinner dolphins (*Stenella longirostris longirostris*) and bottlenose dolphins (*Tursiops truncatus*) in Hawaiian waters have also been recognized as discrete population stocks^42–45^ and evidence suggests that rough-toothed dolphins, Cuvier’s beaked whales (*Ziphius cavirostris*), Blainville’s beaked whales (*Mesoplodon densirostris*), pilot whales and pygmy killer whales (*Feresa attenuata*) additionally represent small island-associated populations^46–50^. The presence of many small resident populations makes Hawaii an especially vulnerable location when considering the potential for disease outbreaks among cetaceans.

A significant challenge in understanding disease impacts and conducting monitoring for disease among Hawaiian cetaceans is that the recovery of cetacean carcasses represents only a very small subset of the animals that are actually dying in the region. Carcass recovery rates of cetaceans that die range between 2 and 25% from other regions, with the high end of 25% for coastal California bottlenose dolphins caveated as likely to be much lower for pelagic dolphins^51,52^. In Hawaiian waters, we estimate carcass recovery rates near 5% for endangered main Hawaiian Islands insular false killer whales and Hawaii Island spinner dolphins where population size and mortality rate estimates are available^39,53^, but expect carcass recovery rates would be substantially lower for a pelagic dolphin such as Fraser’s dolphins that are infrequently sighted and even more infrequently strand. Consequently, a true epizootic event in the central Pacific may only be detectable with the most concerted efforts. This emphasizes the importance of encouraging rapid public reporting of cetacean strandings and highlights the value of examining every single carcass. Additionally, CeMV has very recently been identified from the exhaled breath of two humpback whale groups in Brazil using a highly sensitive RT-qPCR method^54^ that could be applied to non-invasively monitor cetacean populations in the Pacific. Continued disease surveillance of cetaceans is a top priority for understanding potential disease outbreaks that threaten to decimate small island-associated populations in Hawaii and elsewhere.

## Methods

### Fraser’s dolphin stranding and gross necropsy

On January 17^th^, 2018 a stranded Fraser’s dolphin was reported dead on the beach near Olowalu, Maui (20.79345°N, − 156.5811°W). The carcass was recovered and transported to Oahu for necropsy at the University of Hawaii Health and Stranding Laboratory. The necropsy was conducted on the same day and included morphometrics, stomach content examination for prey identification, external and internal gross examination, internal organ weights and extensive formalin-fixed and frozen tissue collections for histopathology and disease screening.

### Prey identification from stomach contents

Cephalopod beaks were identified to the lowest possible taxon using the private reference collection of W.A. Walker and the cephalopod reference collection housed at the Marine Mammal Laboratory, Seattle, Washington. The number of lower beaks present was used to estimate the total number of cephalopod species. Dorsal mantle length and total weights were estimated by measuring lower beak rostral length and then applying the appropriate regression equations. Cephalopod beaks were measured to the nearest 0.1 mm with either an optical micrometer or Vernier calipers and regression equations from the literature were used to estimate prey size and mass for the cephalopod species present^55,56^.

### Immunohistochemistry

Morbillivirus immunohistochemical (IHC) testing was conducted following an in-house developed diagnostic laboratory protocol, as previously described^57^. Epitope retrieval was done using citrate buffer (pH 6.0). Nonspecific binding was blocked with 3% hydrogen peroxide and a commercially available blocking agent (Power Block, Biogenex, San Ramon, CA). The primary antibody was a mouse monoclonal antibody directed against the nucleoprotein of canine distemper virus (#CDV-NP, Veterinary Medical Research and Development, Pullman, WA) but known to cross-react with other morbilliviruses, at a final concentration of 1:400. The secondary antibody was biotinylated horse anti-mouse IgG, rat absorbed (Vector Labs, Burlingame, CA), and the conjugate Streptavidin–horseradish peroxidase (Dako, Carpinteria, CA). The substrate-chromogen system was 3,3′-diaminobenzidine (DAB) (Dako, Carpinteria, CA). Tissue sections were counterstained with Gill’s hematoxylin. Positive control was a CDV-infected formalin-fixed and paraffin embedded Vero cell pellet, while the negative control was similarly treated uninfected Vero cell pellet. Positive and negative controls were included in each IHC run.

### Morbillivirus isolation

Signaling lymphocyte activation molecule (SLAM) or CD150 is a member of the C2 subset of the immunoglobulin superfamily and is expressed on a variety of immune cells from a number of mammalian species. In addition to its function of regulating immune response, it has been shown to be a virus receptor for morbilliviruses. Canine SLAM has been transfected into Vero cells^58^ and this Vero.DogSLAMtag cell line has been used successfully to isolate phocine distemper virus (PDV) from experimentally infected ferrets (*Mustela putorius furo)* and to significantly reduce the incubation time for propagating CeMV in vitro showing its usefulness for morbillivirus isolation^59^. Therefore, Vero.DogSLAMtag cells were chosen to attempt virus isolation of CeMV from frozen tissues collected at necropsy from the Fraser’s dolphin (Table 1). Briefly, tissues were rapidly thawed and two sub samples of approximately 1 g from each tissue were ground in a mortar and pestle with added silica sand. Dulbecco’s modified Eagle’s medium/Ham’s F-12 (DMEM/F-12) plus penicillin 200 IU/mL, streptomycin 200 mg/mL and gentamicin 50 µg/mL (Thermo Fisher Scientific, Nepean, Canada); was added to give a 10% w/v) suspension. Tubes were centrifuged to remove cellular debris for 10 min. (2060 X G), and 500 µl served as inoculum for Vero.DogSLAMtag cells simultaneously seeded into 25 cm^2^ flasks containing 5.0 mL DMEM/F-12 with 4% fetal bovine serum (FBS) to give approximately an 80% confluence of cells. Cell controls were mock infected with diluent. After 24 h incubation at 37^0^ C the media was removed and DMEM/F-12 without serum was added before being returned to the incubator. Flasks were examined daily for signs of CPE passaged weekly, again to give approximately 80% confluence, in DMEM/F12 plus 4% FBS. After 24 h media was changed to DMEM/F-12 without FBS. This procedure was repeated weekly for 1 month at which time the flasks showing no visible CPE were discarded.

Flasks of cells infected with passage one of the isolate were first harvested at 2 days post-infection, then daily from day six until ten days post-infection encompassing all phases of CPE development from initial syncytial development to total monolayer destruction, then processed for transmission electron microscopy.

### Transmission electron microscopy

In order to harvest cells detached from the monolayer and viral particles released into the medium, an amount of primary fixative (modified Karnovsky’s, 2% formaldehyde prepared from paraformaldehyde + 2% glutaraldehyde in 0.1 M cacodylate buffer pH 7.3) equal to that of cell culture medium was added to the flask and left for one hr at room temperature. The liquid was removed, placed in a tube and centrifuged to pellet the cells. Supernatant fluid was removed and the pellet re-suspended in full-strength fixative. The monolayer in the flask was immediately covered with full strength fixative after the removal of floating cells in fixative and incubated for a further 2 h, again at room temperature. The cells were washed with cacodylate buffer, scraped off, pelleted, resuspended in PBS and shipped overnight on ice-packs to UTMB Pathology Electron Microscopy Laboratory. The pellets were washed in cacodylate buffer and kept overnight in 2P + 2G fixative at 4 °C. They were washed in cacodylate buffer, post-fixed in 1% OsO_4_ in the same buffer and further processed as described earlier^60^.

### Morbillivirus detection by RT-PCR

RNA was extracted from frozen samples (i.e. cerebellum, left lung, liver, spleen, left hilar lymph node, mediastinal lymph node and right pre-scapular lymph node) using a commercial kit RNeasy Mini Kit (QIAGEN, Valencia, CA) according to manufacturer instructions. All samples were tested using a reverse transcription polymerase chain reaction (RT-PCR), which were based on consensus degenerate primers (*L* gene) used for the detection of paramyxoviruses that target a conserved region of the RNA dependent RNA polymerase^61^. Briefly, the following steps were used for the RT-PCR: one cycle of 50 °C for 30 min for cDNA synthesis and one cycle of 95 °C for 15 min for denaturing, followed by 40 amplification cycles of 94 °C for 1 min, 45 °C (ResMorHen-F1/ResMorHen-R) for 1 min, 72 °C for 1 min, and a final elongation cycle of 72 °C for 10 min. The PCR products were analyzed on a 1% agarose gel stained with ethidium bromide. Fragments of the expected size (~ 559 bp) were purified using a QIAquick Gel extraction kit (QIAGEN, Valencia, CA) and sequenced in both directions using an ABI 3130 DNA sequencer (Life Technologies, Carlsbad, CA).

The partial *P* gene was sequenced by designing overlapping pairs of primers that were based on the complete genome sequence of a CeMV previously determined from a Mediterranean striped dolphin (DMVSc90-92_Meditteranean; GenBank accession no. AJ608288). RNA samples were subjected to a series of overlapping one-step RT-PCR reactions using a OneStep RT-PCR Kit (QIAGEN, Valencia, CA) as recommended by the manufacturer. The following steps were used for the one-step RT-PCR reactions: one cycle of 50 °C for 30 min for cDNA synthesis and one cycle of 95 °C for 15 min for denaturing, followed by 40 amplification cycles of 94 °C for 1 min for denaturing, annealing (temperature dependent upon the primer pair used) for 1 min, elongation step at 72 °C for 1 min, and a final elongation step at 72 °C for 10 min. The PCR products were then subjected to electrophoresis in 1% agarose gel stained with ethidium bromide. DNA fragments of the expected sizes were purified using a QIAquick Gel Extraction Kit (QIAGEN, Valencia, CA) and was sequenced in both directions using an ABI 3130 DNA sequencer (Life Technologies, Carlsbad, CA).

### Phylogenetic analysis

The phylogenetic analysis was performed based on the 49 partial nucleotide (nt) sequences of the P gene. The nt sequences were aligned in MAFFT 7 using default parameters^62^. The final data set contained 248 characters including gaps. Maximum Likelihood (ML) phylogenetic analysis was performed in IQ-TREE version 1.4.4 (http://iqtree.cibiv.univie.ac.at/) with the Bayesian information criterion to determine the best model fit and 1000 non-parametric bootstraps to test the robustness of the clades. The phylogenetic tree was then edited using FigTree v1.4.2^63^. For the genetic analyses, partial nucleotide sequences of the P (49 morbillivirus sequences with 248 characters including gaps) and L (41 morbillivirus sequences with 559 characters including gaps) genes were compared using the Sequence Demarcation Tool v1.2^64^ with the MAFFT 7 alignment option implemented.
