# Author Correction: Novel cetacean morbillivirus in a rare Fraser’s dolphin (*Lagenodelphis hosei*) stranding from Maui, Hawai‘i

**DOI:** 10.1038/s41598-021-99650-w

**Published:** 2021-10-05

**Authors:** Kristi L. West, Ilse Silva‑Krott, Nelmarie Landrau‑Giovannetti, Dave Rotstein, Jeremiah Saliki, Stephen Raverty, Ole Nielsen, Vsevolod L. Popov, Nicole Davis, William A. Walker, Kuttichantran Subramaniam, Thomas B. Waltzek

**Affiliations:** 1 Hawai‘i Institute of Marine Biology, University of Hawai‘i at Manoa, Kaneohe, HI USA; 2 Human Nutrition Food and Animal Sciences, College of Tropical Agriculture and Human Resources, University of Hawai‘i at Manoa, Honolulu, HI USA; 3 Department of Infectious Diseases and Immunology, College of Veterinary Medicine, University of Florida, Gainesville, FL USA; 4 Marine Mammal Pathology Services, Olney, MD USA; 5 Oklahoma Animal Disease Diagnostic Laboratory, College of Veterinary Medicine, Oklahoma State University, Stillwater, OK USA; 6 Animal Health Center, British Columbia Ministry of Agriculture, Abbotsford, BC Canada; 7 Department of Fisheries and Oceans Canada, Winnipeg, MB Canada; 8 Department of Pathology, The University of Texas Medical Branch, Galveston, TX USA; 9 Pacific Islands Regional Office, National Marine Fisheries Service, Honolulu, HI USA; 10 Marine Mammal Laboratory, National Marine Fisheries Service, Seattle, WA USA

Correction to: *Scientifc Reports* 10.1038/s41598-021-94460-6, published online 09 August 2021

The original version of this Article contained errors.

The authors omitted the below Reference, which is listed below as Reference 37.

37. Duignan, P.J., *et al.* Morbillivirus infection in cetaceans of the western Atlantic. *Vet. Microbiol.* **44**, 241–249. 10.1016/0378-1135(95)00017-5 (1995).

As the Result, in the Discussion section,

“Fraser’s dolphins are a pelagic species that is poorly known from the world’s oceans. There are no previous reports of morbillivirus in Fraser’s dolphins from Hawaiian waters or the greater United States but known morbillivirus testing in this region is limited to seronegative results that were obtained from 10 Fraser’s dolphins that previously mass stranded in Florida in 2003^36^. However, three of four Fraser’s dolphins that had stranded in 1997 and 1999 along the coasts of Argentina and Brazil respectively, had antibodies against DMV^37^. This suggests that morbillivirus is likely endemic in Fraser’s dolphins from the Southwest Atlantic and it is possible that a novel strain of morbillivirus is similarly circulating among this species in the central Pacific. Fraser’s dolphin strandings are extremely rare in Hawaiian waters, with the 2018 individual only the second confirmed stranding of this species in this region. A young Fraser’s dolphin was reported dead off Kauai in an advanced state of decomposition in 2004 and a possible newborn Fraser’s dolphin was reported dead stranded in 1976^15^. With such extreme rarity of stranding, we have not had the opportunity to date to test additional Fraser’s dolphins from the central Pacific for the presence of morbillivirus or morbillivirus antibodies.”

now reads:

“Fraser’s dolphins are a pelagic species that is poorly known from the world’s oceans. There are no previous reports of morbillivirus in Fraser’s dolphins from Hawaiian waters. In the greater United States seronegative results were obtained from 10 Fraser’s dolphins that mass stranded in Florida in 2003 and PMV seropositive results from 11 of 23 mass stranded Fraser’s dolphins in the Gulf of Mexico in 1994^36,37^. Additionally, three of four Fraser’s dolphins that had stranded in 1997 and 1999 along the coasts of Argentina and Brazil respectively, had antibodies against DMV^38^. This suggests that morbillivirus is likely endemic in Fraser’s dolphins from the Gulf of Mexico and the Southwest Atlantic and it is possible that a novel strain of morbillivirus is similarly circulating among this species in the central Pacific. Fraser’s dolphin strandings are extremely rare in Hawaiian waters, with the 2018 individual only the second confirmed stranding of this species in this region. A young Fraser’s dolphin that was reported dead off Kauai in an advanced state of decomposition in 2004 and a possible newborn Fraser’s dolphin was reported dead stranded in 1976^15^. With such extreme rarity of stranding, we have not had the opportunity to date to test additional Fraser’s dolphins from the central Pacific for the presence of morbillivirus or morbillivirus antibodies.”

As a result of the changes, the References have been renumbered.

The original Article has been corrected.

